# Urethral rhinosporidiosis: Prolapsing mass from urethra in a Bangladeshi farmer

**DOI:** 10.1016/j.idcr.2023.e01825

**Published:** 2023-06-16

**Authors:** B.M. Saiduzzaman, Saumitra Chakravarty, Md. Shafiqur Rahman, Mominul Haider, Md. Mominul Islam, Md. Shaleh Mahmud

**Affiliations:** a Department of Urology, Bangabandhu Sheikh Mujib Medical University, Dhaka, Bangladesh; b Department of Pathology, Bangabandhu Sheikh Mujib Medical University, Dhaka, Bangladesh

**Keywords:** Rhinosporidiosis, Urethra, Prolapsing urethral mass, Sporangia, Endospores

## Abstract

Rhinosporidiosis is a chronic granulomatous fungal infection caused by *Rhinosporidium seeberi*. Usual site of infection is the nasal mucosa & nasopharynx. Male urethra is extremely rare site involving this disease. Here we are reporting a rare case where rhinosporidiosis presented as a prolapsing mass from urethra during voiding.

## Introduction

Fungal disease Rhinosporidiosis was first described by Gulleimaro Seeberi in 1900. It is a chronic granulomatous condition usually affecting the nasal mucosa, caused by a fungus Rhinosporidium Seeberi. Ashworth in 1923 described the lifecycle of this fungus and established the name. The organism is now classified under mesomycetozoa as a parasite. Though it is globally distributed but 90% cases are from Asia mainly from South India, Sri Lanka, Pakistan and less than 5% case are from Africa & western countries. The nasopharynx and nasal mucosa commonly (70%) affected in this disease but it may infects conjunctiva, lacrimal sac, lip, palate, skin, larynx, trachea, bronchi, vagina and vulva are the other sites which may be affected. Urethra may be involved but it is extremely rare, only few cases reported in literature till date and they are mostly from India. Here we report a case of urethral rhinosporidiosis prolapsing from external urethral meatus during voiding.

## Case report

**Fig. 1:**
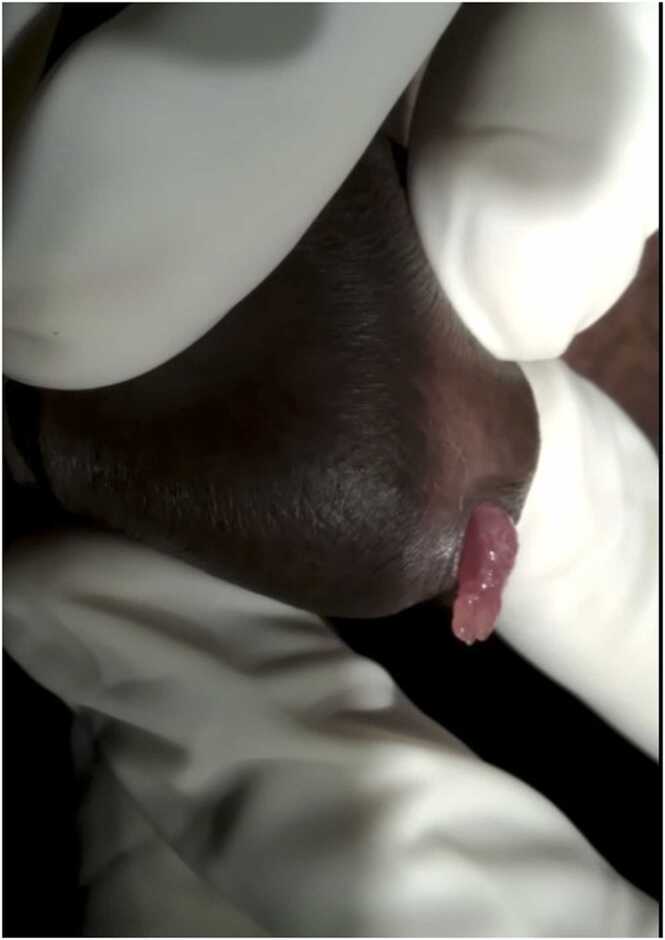
Protruding mass when urethral meatus is everted.

A 40-year-old man hailing from a rural area of Bangladesh, farmer by occupation presented with the complaint of some fleshy red mass coming out per urethra during voiding for last 4 months. He also complains burning sensation during micturition for same duration. He noticed a red mass like a fish tail, protruded per urethra during voiding and it disappears when voiding is completed. The size of protruding mass also increased gradually from size of a very small grain to the presenting size of 2 cm × 1 cm. It is visible when urethral meatus is everted (Fig. 1). He had no history passage of blood with urine or contact bleeding. There is no other significant past medical or surgical history.

On Physical examination, he had normal external genitalia with an elongated fishtail reddish mass measuring 2 cm from the tip to the base of the mass, when urethra is manually everted or glans is compressed. Mass is soft, non-tender and bleeds on touch. No inguinal lymphadenopathy was found. His hematological and renal biochemical parameters were normal. Ultrasonography of KUB region was normal.

**Fig. 2:**
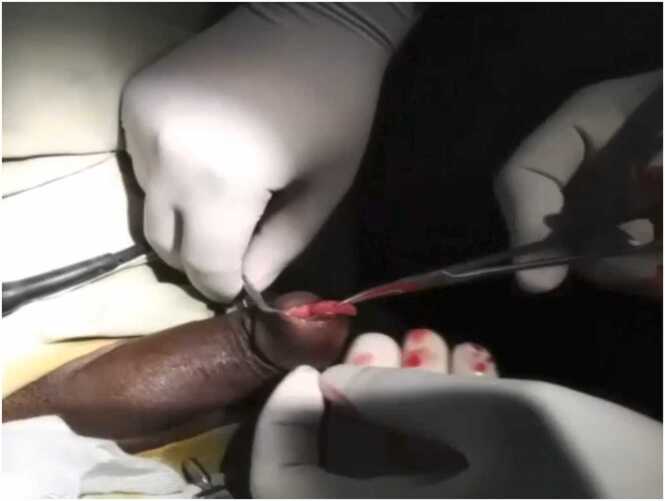
Polypoidal elongated mass arising from fossa navicularis.

Under Subarachnoid block anesthesia, mass was excised with low current electro-coagulation of the base and urethra was repaired on the operated area. A pedunculated red fleshy mass arising from the ventral aspect of the urethra, about 2 cm proximal to the external urethral meatus was found (Fig. 2). Then urethrocystoscopy was done and it revealed rest of the urethra and urinary bladder to be normal. Meatotomy wound was closed leaving per urethral catheter.

**Fig. 3:**
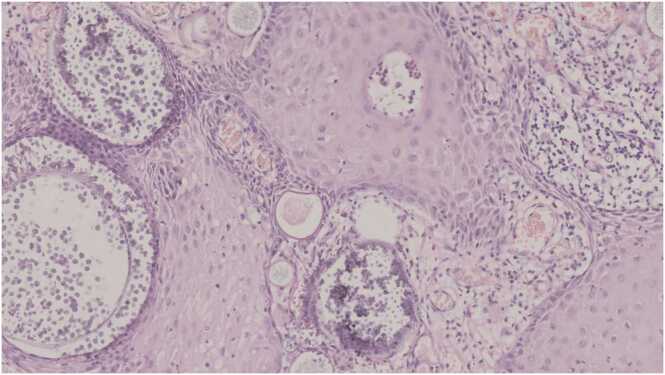
Photomicrograph shows multiple sporangia with endospores (HE, x20).

**Fig. 4:**
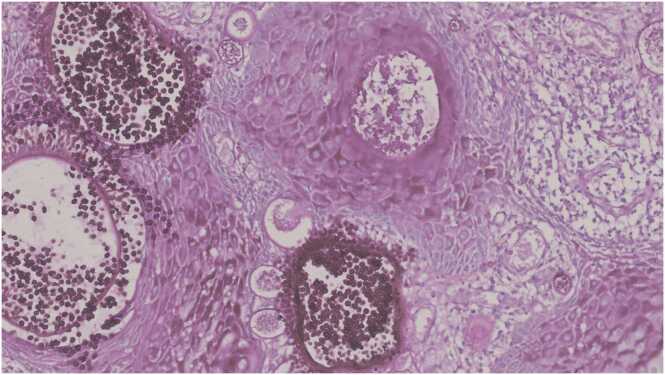
Photomicrograph shows multiple sporangia with endospores (PAS, x20).

On histopathological examination of the excised mass it was found that the sections of the submitted specimen revealed many large thick sporangia with numerous endospores accompanied by mixed inflammatory infiltrate (Fig. 3, Fig. 4). Final Diagnosis was urethral rhinosporidiosis.

**Fig. 5:**
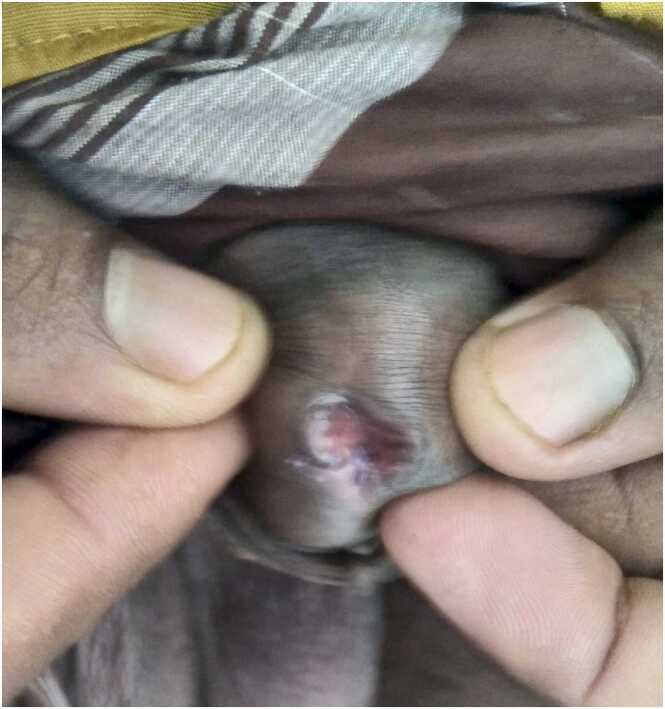
No recurrence is seen after 2.5 years.

Physical examination and urethrocystoscopy was done at 3 month follow up. Patient was doing well 2.5 years without any recurrence (Fig. 5). Histopathological photomicrographs and virtual slides of this case are publicly available [1].

## Discussion

Rhinosporidiosis is a chronic granulomatous cutaneous disease commonly found in India, Sri Lanka, Pakistan, and Africa; rarely seen in western world [2]. Most of the cases the patients presented from the rural area and poor socioeconomic status [2], [3]. It is a painless infectious condition caused by a protoctistan mesomycetozoa, *Rhinosporidium seeberi*, which affects predominantly the mucous membrane of the nose and nasopharynx. Previously categorized as a fungus, recent molecular biological analysis of the ribosomal DNA of organism has confirmed beyond doubt its categorization as member of a group of novel aquatic parasites that flourish in hot and humid climate. It’s mode of transmission is not clearly known till now. Contaminated water, dust and soil are the likely source of infection and rural background of the patients seems to substantiate this [3], [4]. It is not contagious there is no evidence of transmission of the disease from human to human or animal to human till now [3], [5].

Usually, the lesion presents as a discrete, friable, painless slow growing polypoidal pedunculated or sessile masses which are highly vascular and bleeds on touch [3], [4]. Nasal mucosa, Nasopharynx (70%) and conjunctiva is the most common site of infection, but other rare sites are larynx, maxillary antrum, and skin of limbs, lacrimal sac, urethra, vagina, parotid duct, bone and rectum [5]. Urethral involvement is very rare and only few cases are reported in the literature, mostly from India [3], [5]. Typical clinical features of the lesion and strong suspicion lead to the diagnosis (Table 1).

**Table 1:** Summary of previously reported cases of urethral rhinosporidiosis in English medical literature.

Ref.	No. of case (s)	Age (years) at presentation	Sex	Country	Presentation	Treatment	Outcome (at least 3 months follow-up)
Dhayagude et al [6]							
Palaniswamy and Bhandari [7]	1	27	M	India	Bloody ejaculate, thin urinary stream, occasional per urethral bleeding and a mass projecting from the urethral meatus with a central fistuala	Complete excision of mass and fistula tract and marsupialization of 5 cm of the distal urethra followed by plan for urethroplasty over next 2 years	Recurrence
Sasidharan et al. [5]	25	15–37	M	India	Polypoid to tumor-like mass measuring 2–3 cm developed over 3–15 months	Excision	Recurrence in 7 cases
	2	Mid-forties	F				
Qureshi et al. [8]	1	48	M	India	Fleshy mass from external urethral meatus for 10 years	Complete endoscopic excision with base electrocautery	No recurrence
Hanchanale et al. [9]	1	26	M		Per urethral bleeding for 4 months, cystouroscopically visible fleshy granular vascular polyp	Resection with base diathermy	No recurrence
Pal et al. [10]	1	27	M	India	Pinkish mass protruding from external urethral meatus for 2 years, following excision of similar lesion 7 years back	Transurethral resection of the mass using low coagulation current	No recurrence
Pal et al. [11]	5	24	M	India	Pinkish polypoid mass on external urethral meatus without obstruction	Excision with base diathermy	Not mentioned
		36	M	India	Pinkish mass having granular surface beneath foreskin with postcoital bleeding for 3 months	Ventral meatotomy followed by excision with base diathermy	Not mentioned
		43	M	India	Slow-growing cancer-like mass on glans penis for 2 years	Excision with base diathermy	Not mentioned
		27	M	India	Pinkish fleshy mass protruding from urethra during micturition	Ventral meatotomy followed by excision with base diathermy	Not mentioned
		23	F	India	Tongue-like fleshy projection from posterior lip of urethra, initially suspected urethral leiomyoma	Excision with base diathermy	No recurrence but developed meatal stenosis and undergoing self-meatal dilatation
Azad et al. [12]	1	26	M	Pakistan	Polypoid lesion from external urethral meatus with dysuria and bloody discharge for 3 months	Complete resection	No recurrence
Mallick et al. [13]	1	50	M	India	Multiple mass-like lesion involving nose, perinium and urethra for 3 years	Excision with base electrocautery, followed by Dapsone administration	No recurrence
Subitha et al. [14]	1	40	M	India	Fleshy growth from urethral orifice, recurred 5 years after excision	Not mentioned	Not mentioned
	1	60	M	India	Protruding polypoid urethral growth	Not mentioned	Not mentioned
Bandyopadhyay et al. [15]	1	50	M	India	Disseminated with nasal obstruction (bilateral, epistaxis), involvement of skin, subcutaneous tissue, wrist, bone, buttock, penis and glans penis near urethra for 20 years	Wide local excision with base diathermy and glans penis amputation	Recurrence
Pandey et al. [16]	1	61	M		Recurrent passage of flakes in urine and protruding mass from urethral meatus for 8 months	Excision	No recurrence
Seth et al. [17]	1	32	M	India	Pinkish slow-growing polypoid mass on glans penis near external urethral meatus for one year	Excision with base diathermy	No recurrence
Das et al. [18]	1	26	M	India	Disseminated with nasal obstruction (bilateral, epistaxis), involvement of skin, subcutaneous tissue, bone, penis and urethra for 15 years	Wide local excision with base electrocautery	Recurrence
Ahmed et at. [19]	1	45	M	Bangladesh	Polypoidal lesion at EUM	Resection under spinal anesthesia	No recurrence
Reddy et al. [20]	1	37	M	India	Polypoidal lesion at EUM	Surgical excision, followed by diathermy coagulation of the base	Not mentioned

There were at least four more cases in published English literature which were not available online or through library archives. Total 50 cases. Among them, one case found from Bangladesh in 2015.

Histopathological examination is the only way to confirm the diagnosis [3], [4], [5]. It reveals chronic inflammatory cell infiltrate composed of plasma cells, lymphocytes along with foreign body giant cells surrounding characteristic sporangia, seen as globular cysts of various sizes at various stages of maturity lined by well-defined chitinous wall [2], [3], [4], [5]. Inside the sporangia are present numerous endosporse. In our case diagnosis was made by characteristic histopathology showing sporangia and endospores with mixed inflammatory cell infiltrate. Though the disease has benign course usually and remain localized, two fatal cases with disseminated rhinosporidiosis has been reported [5].

Surgical excision under spinal anesthesia followed by electro-coagulation of the base is the preferred method of treatment [3], [5] same treatment was given to our case. Recurrence has been documented up to 25% due to inadequate resection [3]. Sometimes, partial amputation of the penis may be required, due to extensive involvement of the glans [3]. Systemic amphotericin B and Dapsone has been used with inadequate response [4].

## Funding

This research received no specific grant from any funding agency in the public, commercial, or not-for-profit sectors.

## Ethical approval

All necessary measures have been taken to ensure ethical treatment of the patient and their data.

## Consent

Written informed consent was obtained from the patient for publication of this case report and accompanying images.A copy of the written consent is available for review by the Editor-in-Chief of this journal on request.

## CRediT authorship contribution statement

**B.M. Saiduzzaman**: Conceptualization, Writing – original draft, Data analysis, Study design, Visualization, Validation. **Saumitra Chakravarty:** Data collection, Writing – review & editing, Data analysis, Data curation, Visualization, Validation. **Md. Shafiqur Rahman:** Supervision, Resources. **Mominul Haider:** Conceptualization. **Md. Mominul Islam:** Investigation, Data curation. **Md. Shaleh Mahmud:** Investigation, Data curation.

## Declaration of Competing Interest

None of the authors have any conflict of interest regarding this work.
